# Leadless pacemaker implantation in patients with bioprosthetic tricuspid valve replacement: A case report and review of the literature

**DOI:** 10.1016/j.hrcr.2024.06.014

**Published:** 2024-06-21

**Authors:** Evan Aders, John S. Strobel

**Affiliations:** ∗ Indiana University School of Medicine, Indianapolis, Indiana; † Indiana University Health, Bloomington Hospital, Bloomington, Indiana

**Keywords:** Leadless pacemaker, Bioprosthetic tricuspid valve, Atrioventricular block, Infective endocarditis, Permanent pacemaker

**Key Teaching Points**

• Bioprosthetic tricuspid valve (BTV) replacement is associated with postoperative atrioventricular block, requiring some form of permanent pacing in many patients.
• Based on our review of the limited number of published case reports and case series, leadless pacemaker (LPM) insertion can be performed safely following BTV replacement, with no reported increased procedural risk, no adverse effects on the BTV, fewer associated incidences of infection, and excellent long-term pacing function when compared to traditional pacing systems with leads.
• LPMs are an option for patients in need of permanent pacing status post BTV replacement.

## Introduction

In patients undergoing bioprosthetic tricuspid valve (BTV) replacement, the incidence of atrioventricular (AV) block and need for pacemaker implantation is high.^1^, ^2^, ^3^ In these patients, placement of transvenous leads is discouraged since leads crossing the BTV may lead to valve damage or dysfunction; furthermore, in many of these patients, the indication for BTV is frequently recent or ongoing infection, and placement of transvenous leads may increase this risk. Finally, placement of transvenous leads adds risks of pneumothorax, hemothorax, and cardiac perforation, as well as chronic postoperative complications including increased risk of deep vein thrombosis, lead dislodgement, and lead fracture, in addition to the aforementioned risk of infections.^4^, ^5^, ^6^

Epicardial lead placement is an alternative approach that is often used to circumvent the BTV, but it also comes with several unique drawbacks.^4^ Epicardial leads traditionally have been shown to have a shorter longevity related to elevated pacing thresholds, which may be important in the population of BTV patients, who are often younger. In addition, placement of epicardial leads is more invasive.

Leadless pacemakers (LPMs) have recently been introduced as an alternative to traditional transvenous lead placement, but the safety and efficacy of these devices in patients with BTV replacements has not been extensively studied. Questions may exist regarding procedural safety, acute or long-term damage to the valve, infection risk, and efficacy of pacing. Reassuring data exist; however, relatively few cases are reported in the literature.^6^, ^7^, ^8^, ^9^, ^10^, ^11^

## Case report

A 30-year-old woman with a history of intravenous drug abuse presented in August 2019 with sepsis. Echocardiogram demonstrated vegetations of the tricuspid and mitral valves. Following antibiotic treatment, follow-up echocardiogram in December 2019 demonstrated that the vegetations had resolved. Sepsis related to endocarditis recurred in October 2020. BTV was required in December 2020 but was complicated by the development of complete heart block. A dual-chamber pacemaker using a transvenous atrial lead and epicardial ventricular leads was implanted.

She presented to our clinic in September 2022 for follow-up. At that time, she reported symptoms of device pulsation and chest tightness, and had a ventricular threshold of 3 V at 1 ms. Though it was recognized that battery longevity would be reduced, the pacing outputs were increased to provide an appropriate safety margin, since the patient was pacemaker dependent. At that time, the patient was informed that when pacemaker replacement was required, LPM would be advised.

She then presented again in May 2023 and the ventricular threshold had risen to 4.0 V at 0.5 ms. LPM was recommended at this time. In June 2023, a Medtronic Micra AV LPM was implanted. There was no difficulty in advancing the pacemaker delivery system across the BTV. Initial threshold was 1.5 V at 0.5 ms, although a rise in threshold was noted following removal of the retention suture. At follow-up 1 month later, however, the threshold was improved (0.5 V at 0.24 ms); and at the most recent device check in December 2023 the threshold remained stable (0.5 V at 0.24 ms). In addition, echocardiography performed in January 2024 demonstrated normal LV systolic function with an ejection fraction of 61% and moderate regurgitation of the BTV, unchanged from prior echo in October 2022.

## Discussion

In a study performed by Koplan and colleagues^2^ involving the development of a preoperative risk score to predict need for permanent pacing after cardiac valve surgery, it was found that valvular surgery involving the tricuspid valve (TV) was one of the strongest independent predictors for postoperative permanent pacemaker placement; specifically, the study estimated a 3.7-fold increase in the need for permanent pacemaker in these patients. To address this, standard transvenous leads can be placed across the BTV into the right ventricle (RV), though this may potentially cause dysfunction of the BTV in a manner similar to that seen in native TVs. For this reason additional approaches had been developed, including the surgical placement of an epicardial ventricular lead, as seen in our patient. Despite their drawbacks, there remains some utility for epicardial leads in modern practice, such as for patients such as ours for whom avoiding BTV damage is desirable. Unfortunately, while one advantage of epicardial leads is circumventing the BTV, epicardial lead placement is inherently more invasive than transvenous lead placement owing to its requirement for general anesthesia and thoracotomy; additionally, lead longevity is less with epicardial leads with a greater incidence of exit block and higher thresholds on follow-up when compared to transvenous leads. The use of epicardial suture electrodes has been an improvement over screw-in leads. These disadvantages and limitations were clear in our patient, who was suffering the physical consequences of rapidly increasing thresholds of her epicardial lead as well as impaired battery life owing to lead inefficiency.

More recent data support the use of LPMs in patients with symptomatic AV block as opposed to traditional forms of dual-chamber pacing.^12^ The introduction of LPM is relatively new and data involving large populations exist, proving its efficacy in patients with native TVs when compared to traditional dual-chamber pacing; however, data on its efficacy in patients with BTVs are somewhat limited.

In patients with BTV, several questions exist. First, in this population of patients, is LPM safe and feasible? Specifically, are the acute and/or long-term risks increased in this already high-risk population? Additionally, can the LPM delivery system be passed into the RV without damage to the BTV but with acceptable pacing and sensing parameters? And can the LPM be placed early after BTV? Finally, are there unique long-term risks of LPM implant in this population, such as infection, valve dysfunction, or elevated pacing thresholds?

Kerwin and colleagues^7^ reported a single case as early as 2015 involving a patient status post mitral valve (MV) and TV replacement with atrial fibrillation and high-degree AV block with symptomatic bradycardia, for whom LPM placement was recommended over transvenous pacing owing to a newly implanted BTV. Del Corral and colleagues^8^ and Boveda and colleagues^9^ also reported single cases involving patients with histories of BTV and progressively failing epicardial leads, similar to our patient, for whom LPM was proposed.^8^^,^^9^ In these reports, LPM was performed safely, without a significant increase in risk of procedural duration or complications; pacing parameters were acceptable; and no adverse effects on the BTV were reported.

Morani and colleagues^10^ reported on 2 patients for whom LPM implantation was performed safely, also after failure of epicardial leads. Some concern was reported regarding the risk of possible mechanical contact between the LPM delivery system and the valve structure, but the group reports careful handling of the delivery system, proper use of imaging in the form of radiography oblique views, and thoughtful selection of deployment site can result in successful, atraumatic implantation. Follow-up for both patients revealed acceptable electrical parameters, satisfactory RV function, and no interference of the LPM with the BTV after 6 months.^10^

Afzal and colleagues^11^ compared LPM implantation between a group of patients with no prior TV intervention and a group of patients with prior TV intervention, including both BTV replacement and repaired TV. In the latter group, there were 12 patients with BTV specifically. Various adjunctive techniques were reportedly used to facilitate some of the procedures, such as intracardiac echocardiography and low-profile (5F deflectable) electrophysiology catheters. These techniques were employed when ensuring alignment of the LPM delivery system crossing though the BTV, but resulted in the same outcomes as the procedures that did not use these adjunctive techniques for assistance. Although the procedural duration was increased when compared to LPM implantation in patients without prior TV intervention, no differences in long-term outcomes were seen between the 2 groups studied.^11^

Theis and colleagues^6^ presented a retrospective analysis of 14 patients in whom recent TV repair or replacement had been performed. In their report, the LPM was implanted a mean of 4.76 ± 1 days after BTV replacement with a low mean number of attempted deployments (1.6 ± 1.26), without any residual damage, showing LPM is a feasible option in BTV recipients even in the very early postoperative window. Additionally, the analysis identified 2 patients who received their LPM shortly after resolution of MV endocarditis who had no signs of recurrence after follow-up, suggesting LPM implantation after MV or TV surgery owing to endocarditis as a viable solution.^6^ Beurskens and colleagues^13^ support this finding with a larger study that showed no LPM infections at 20-month follow-up status post LPM implantation within 2–7 days of extraction of entire infected pacing systems. With this in mind, LPMs may certainly provide the added benefit of reduced risk of long-term device infection. Because patients who present with needs such as our patient’s often have a history of bacteremia and infective endocarditis, minimizing surface area of implanted devices that serve as a nidus for infection should be prioritized. Of note, El-Chami and colleagues^14^^,^^15^ confirmed the absence of LPM infections in clinical trials, even in the setting of bacteremia. LPMs provide this advantage over traditional pacing systems via multiple proposed mechanisms, including smaller surface area, tendency for encapsulation of the device by endocardial tissue, and absence of subcutaneous device housing.^1^

One potential concern related to first-generation LPMs relates to the lack of AV synchrony, since first-generation devices only provide atrial sensing without the ability to pace. In patients with concomitant sinus node dysfunction, AV synchrony will likely be much less than could be provided by an epicardial pacing system. It is the authors’ experience that lack of AV synchrony uncommonly has significant clinical effects. It is expected that future generations of LPMs will improve on this, either by improved atrial mechanical sensing or by the addition of a leadless atrial device in combination with the ventricular device.

## Conclusion

Based on our review of the limited number of published case reports and case series, LPM insertion can be performed safely following BTV replacement with no reported increased procedural risk, no adverse effects on the BTV, fewer associated incidences of infection, and excellent long-term pacing function when compared to traditional pacing systems with leads; therefore, LPMs should be considered an option for patients in need of pacing status post BTV replacement owing to infective endocarditis or other etiologies.

## Disclosures

None (EA); Compensation for leadless pacemaker implantation as part of employment (JS).
